# CBL-b E3 ligase-mediated neddylation and activation of PARP-1 induce vascular calcification

**DOI:** 10.1038/s12276-024-01322-y

**Published:** 2024-10-01

**Authors:** Duk-Hwa Kwon, Sera Shin, Yoon Seok Nam, Nakwon Choe, Yongwoon Lim, Anna Jeong, Yun-Gyeong Lee, Young-Kook Kim, Hyun Kook

**Affiliations:** 1 Department of Pharmacology, Chonnam National University Medical School, Hwasun, Jeollanamdo Republic of Korea; 2 Basic Research Laboratory for Vascular Remodeling, Chonnam National University Medical School, Hwasun, Jeollanamdo Republic of Korea; 3 BK21 plus Center for Creative Biomedical Scientists, Chonnam National University, Gwangju, Republic of Korea; 4 Institute of Well-Aging Medicare & CSU G-LAMP Project Group, Chosun University, Gwangju, Republic of Korea; 5 Department of Biochemistry, Chonnam National University Medical School, Hwasun, Jeollanamdo Republic of Korea

**Keywords:** Neddylation, Calcification

## Abstract

Vascular calcification (VC) refers to the accumulation of mineral deposits on the walls of arteries and veins, and it is closely associated with increased mortality in cardiovascular disease patients, particularly among high-risk patients with diabetes and chronic kidney disease (CKD). Neuronal precursor cell-expressed developmentally downregulated protein 8 (NEDD8) is a ubiquitin-like protein that plays a pivotal role in various cellular functions, primarily through its conjugation to target proteins and subsequent relay of biological signals. However, the role of NEDDylation in VC has not been investigated. In our study, we observed that MLN4924, an inhibitor of the NEDD8-activating E1 enzyme, effectively impedes the progression of VC. LC‒MS/MS analysis revealed that poly(ADP‒ribose) polymerase 1 (PARP-1) is subjected to NEDD8 conjugation, leading to an increase in PARP-1 activity during VC. We subsequently revealed that PARP-1 NEDDylation is mediated by the E3 ligase CBL proto-oncogene B (CBL-b) and is reversed by NEDD8-specific protease 1 (NEDP-1) during VC. Furthermore, the CBL-b C373 peptide effectively mitigated the inactive form of the E3 ligase activity of CBL-b, ultimately preventing VC. These findings provide compelling evidence that the NEDD8-dependent activation of PARP-1 represents a novel mechanism underlying vascular calcification and suggests a promising new therapeutic target for VC.

## Targeting PARP-1 via NEDDylation: a novel VC therapeutic approach

Vascular calcification, a condition where calcium deposits in blood vessels, can increase heart disease risk. Researchers studied a process called neddylation, which modifies proteins, to see if it affects this calcium buildup. They experimented on cells and mice using various techniques like chemical treatments and gene alteration. They found that a protein, NEDD8, when joined to another protein, PARP-1, encourages calcium deposition in blood vessels. By preventing NEDD8 from joining to PARP-1 with a specific inhibitor, they reduced calcium buildup in cells and mice. They also found that an enzyme, Cbl-b, helps NEDD8 attach to PARP-1, suggesting a new way to prevent vascular calcification. Results suggest that stopping neddylation, particularly the joining of NEDD8 to PARP-1, can significantly reduce vascular calcification. This discovery could lead to new treatments for vascular calcification and related heart conditions.

This summary was initially drafted using artificial intelligence, then revised and fact-checked by the author.

## Introduction

Vascular calcification pertains to the abnormal accumulation of calcium within the walls of blood vessels, leading to a reduction in their elasticity and functionality, as well as an elevated risk of cardiovascular events such as heart attack and stroke^1^. This process is intricate and influenced by multiple factors, often originating from various underlying conditions, including aging, diabetes, chronic kidney disease, and atherosclerosis^2^. Vascular calcification manifests in two distinct forms: intimal and medial calcification. Intimal calcification occurs within the inner layer of blood vessels and is closely associated with atherosclerosis^3^, whereas medial calcification affects the middle layer of vessels and is linked to aging and conditions such as chronic kidney disease^4^. Currently, treatment options for vascular calcification are limited, primarily in terms of the management of the causes that contribute to its development.

NEDD8, which stands for neural precursor cell expressed developmentally downregulated 8, is a small protein similar to ubiquitin. It regulates the activity and stability of specific proteins through a process known as neddylation^5,6^. Like ubiquitination, NEDD8 binds to its target proteins by forming an isopeptide chain between its C-terminal glycine residue (Gly76) and a lysine residue on the target protein. This attachment occurs through a series of enzymatic reactions involving the NEDD8-activating enzyme E1, the NEDD8-conjugating enzyme E2, and the NEDD8-ligase enzyme E3^6^. Importantly, this process is reversible, and NEDD8 can be detached from its targets by a family of enzymes known as NEDD8-specific proteases (NEDP1)^7,8^. NEDD8 plays a pivotal role in a variety of cellular processes, including cell cycle regulation, the DNA damage response, and protein degradation in diverse tissues^6,9,10^. Targeting the NEDD8 pathway has emerged as a potential therapeutic strategy for cancer treatment^11,12^. Several small molecule inhibitors of the NEDD8-activating enzyme and NEDD8-conjugating enzyme have been developed, such as MLN4924/Pevonedistat^11,13,14^ and UBE2M-DCN1 inhibitors^15^. These inhibitors are currently undergoing evaluation in clinical trials, indicating significant development in the field of cancer therapy^16–19^.

Poly(ADP‒ribose) polymerase-1 (PARP-1) is a key member of the PARP family, which comprises 18 members and contributes to nearly 90% of cellular PARP activity^19,20^. PARP-1 plays a pivotal role in various cellular processes, including DNA damage repair, transcription, and cell death signaling^20,21^. Numerous studies have provided insights into the multifaceted roles of PARP-1, and its influence extends to a wide array of diseases. These include neurodegenerative disorders^22^, chronic inflammation^23^, cardiovascular diseases^24^ and cancer^25^. Several studies have also demonstrated that PARP-1 initiates the osteogenic transition of vascular smooth muscle cells (VSMCs) by upregulating pivotal transcription factors such as RUNX2 and NF-κB^26,27^. Inhibition of PARP-1 has been shown to have protective effects against mineralization and calcification. Notably, the activity of PARP-1 is influenced by oxidative stress and DNA damage, which, in turn, promote vascular calcification^28,29^. Our research revealed a novel association between NEDD8 and PARP-1 under VC conditions. Despite the recognized involvement of PARP-1 activity in osteogenic calcification, the precise regulatory mechanisms governing its activity during the progression of VC remain to be elucidated.

In the present study, we investigated the significance of NEDD8 in the context of vascular calcification. Our investigation unequivocally revealed that NEDD8 plays a pivotal role in the promotion of vascular calcification, and conversely, the inhibition of NEDD8 serves as a protective measure against this process. Among the conditions conducive to vascular calcification, our observations revealed the activation of PARP-1 due to the interaction facilitated by NEDD8 polychains. This interaction is mediated through the E3 ligase activity of Cbl-b. Our findings suggest that Cbl-b orchestrates the process of PARP-1 neddylation, consequently modulating its activity during the course of vascular calcification.

## Materials and methods

All experimental procedures were approved by the Chonnam National University Medical School Research Institutional Animal Care and Use Committee and followed the National Institutes of Health *Guide for the Care and Use of Laboratory Animals* (NIH Publication No. 8023, revised 1978).

### Induction of vascular calcification

RVSMCs and A10 cells were cultured in growth medium, which was changed to calcification medium containing 2 mM or 4 mM inorganic phosphate (pH 7.4) for 3 days or 6 days. The medium was changed every 2 days. Calcium deposition in the VSMCs was determined after washing with 1x PBS.

Wild-type C57BL/6 male mice were subjected to induction of vascular calcification via the administration of vitamin D_3_ as described previously^30,31^. Vitamin D_3_ (cholecalciferol) in 70 μl of absolute ethanol was mixed with 500 μl of Cremophor (Alkamuls EL-620, Sigma St. Louis, MO, USA) for 15 min at RT and then combined with 6.2 mL of sterilized water containing 250 mg of dextrose for 15 min at RT. The mice were injected with a dose of vitamin D_3_ (5 × 10^5^IU kg^−1^day) subcutaneously for 3 days and maintained for 6 days to induce vascular calcification. The animals were killed by the inhalation of CO_2_ in a chamber, and the aortas were isolated. All experiments were performed with male mice at 8–10 weeks of age. All animal procedures were reviewed and approved by the Institutional Animal Care and Use Committee of Chonnam National University (CNU-IACUC-HA-2020-17 and CNU-IACUC-H-2021-39).

### Measurement of calcium deposition

The A10 cells and tissues were decalcified with 0.6 N HCl at 4 °C for 24 h. The calcium content of the HCl supernatants was determined colorimetrically via a QuantiChrom calcium assay kit (QuantiChromTM Calcium Assay Kit, BioAssay Systems, Hayward, USA) according to the manufacturer’s instructions. Briefly, 5 μL of each sample was transferred to a 96-well plate, and 200 μL of working reagent was added (1:1, solutions A and B). The mixed samples were briefly incubated for 3 min, and the absorbance was measured at 570 nm using an ELx808 absorbance reader (BTELX808, BioTek Instruments, Winooski, VT, USA). Next, the cells were washed 3 times with 1 × PBS and lysed with 0.1 N NaOH/0/1% SDS to extract proteins. The calcium content was then normalized to the total protein amount, whereas that of the tissues was normalized to the dry weight of the tissue.

### Alizarin red S staining and quantification

The A10 cells were washed with 1x PBS and fixed with 10% formalin for 30 min at RT. After being washed three times with distilled water, the cells were stained with 40 mM alizarin red S solution (pH 4.2, Sigma‒Aldrich) for 30 min at room temperature and washed with distilled water to remove nonspecific staining. For quantification of alizarin red S staining, the stained cells were destained with 10% cetylpyridinium chloride (CPC) in 10 mM sodium phosphate buffer (pH 7.4) for 30 min at room temperature. The absorbance was then measured at 450 nm, and the values were normalized to those of a 10% CPC standard solution.

To determine arterial calcification, the aorta samples were collected and fixed with 10% formalin overnight at 4 °C. The arterial tissue sample was placed in 2% KOH overnight at RT and stained with 0.3% alizarin red S in 1.6% KOH for 2 days at RT. After staining, the tissue was placed in preservation solution containing glycerin, 70% ethanol, and benzyl alcohol (2:2:1 ratio) for 3 h at room temperature, followed by a 10 min incubation at 37 °C.

### Proximity ligation assay (PLA)

A PLA was performed via the Duolink^®^ In Situ Red Starter Kit Mouse/Rabbit (Sigma‒Aldrich) according to the manufacturer’s instructions. Before the PLA, the cells were fixed with 4% formaldehyde for 60 min and washed 2 times with 1x PBS. The cells were permeabilized with 0.2% Triton X-100 in PBS for 10 min and then blocked with Duolink blocking solution in a humidified chamber for 60 min at 37 °C. The cells were incubated overnight with anti-PARP-1 and anti-NEDD8 antibodies at 4 °C, followed by treatment with Duolink PLA plus/minus probes for 60 min in a humidified chamber at 37 °C. After the samples were washed 2 times with 1x wash buffer A, PLA signals were generated following the reaction with ligation for 30 min and subsequent amplification for 90 min at 37 °C. The slides were mounted with ProLong™ Diamond Antifade Mountant with DAPI (Thermo Fisher) and photographed via confocal fluorescence microscopy (DE/LSM700, Carl Zeiss Microscopy).

### Administration of MLN4924, si-Cbl-b or Cblb C373 peptide to mice

MLN4924 (10 mg kg^−1^ per day), Cbl-b C373 (1 mg kg^−1^ per day), vehicle or scramble was intraperitoneally injected into the mice. For siRNA injections, 50 µg of siRNA was diluted in 10% glucose, mixed with in vivo jetPEI (polyplus-transfection SA, Illkirch, France) also diluted in 10% glucose, incubated for 15 min at RT, and then vortexed briefly. si-Cbl-b (50 μg per day) or scramble was injected into the tail vein of the mice after equilibration at RT. All of these reagents were administered at the same dose every 2 days for 6 days after vitamin D_3_ injection until the animals were sacrificed.

### PARP-1 activity assay

The cells and tissues were lysed with 0.5% NP-40 solution to extract protein and incubated with anti-PARP-1 overnight at 4 °C. Then, PARP-1 activity was measured via a colorimetric assay kit, following the manufacturer’s protocol. Briefly, a histone-coated plate was activated with 1x PARP buffer for 30 min at RT. After removing the 1x PARP buffer, samples and the PARP-HSA enzyme were added to the wells of the plate, which was subsequently incubated for 10 min at room temperature. The 1X PARP cocktail buffer containing the PARP cocktail was prepared, activated DNA was distributed into the wells and incubated at RT for 1 h, and Strep-HRP working solution was added after the samples were washed 2 times with 1X PBS + 0.1% Triton X-100 (200 μL/well), followed by 2 washes with 1X PBS. The wells were incubated with prewarmed TACS-Sapphire in the dark for 15 min at RT, 0.2 M HCl was added to stop the reaction, and the absorbance at 450 nm was read using an ELx808 absorbance reader (BioTek Instruments).

### Boronate pull-down assay

To detect poly(ADP) ribosylation polymers, boronate-affinity methods were employed with modifications as previously described^32^. Briefly, cells or tissues were lysed via 0.5% NP-40 buffer composed of 50 mM Tris (pH 8.0), 150 mM NaCl, 1 mM EDTA, 1 mM DTT, 1 mM phenylmethylsulfonyl fluoride, 1 mM Na_3_VO_4_, and 1 μg/mL each of leupeptin, pepstatin, and aprotinin and supplemented with 20 nM PARG inhibitor (PDD00017273). The lysates were then mixed with 70 µL of m-aminophenylboronic acid agarose beads and incubated overnight at 4 °C. After incubation, the beads were washed twice with lysis buffer. Proteins eluted from the beads were subsequently analyzed by western blotting.

### Liquid chromatography‒mass spectrometry analysis

NEDD8-conjugated proteins were identified via peptide precipitation and LC‒MS/MS analysis. RVSMCs were treated with high concentrations of phosphate and MLN4924 (1 μM) for 6 days. The extracted proteins were immunoprecipitated with anti-NEDD8 overnight at 4 °C, and the NEDD8-conjugated proteins were subsequently separated on gradient SDS‒PAGE gels (8%-15%) and visualized via Coomassie brilliant blue staining. Specific bands in the Pi-treated lanes were detected, but these bands were diminished by MLN4924 treatment, and LC‒MS/MS was subsequently performed.

Nano LC‒MS/MS analysis was performed at the Korea Basic Science Institute (Biomedical Omics Research Center, Ochang, Korea). The gels were destained with 50% acetonitrile containing 10 mmol of NH_4_HCO_3_ and then rinsed a few times with distilled water to stop the destaining reaction. The gels were incubated with 10 mmol of dithiothreitol and 100 mmol of ammonium bicarbonate at 56 °C to reduce the protein content, followed by the addition of 100 nmol of iodoacetamide to alkylate the cysteines. The gels were then washed with three volumes of distilled water by vortexing and completely dried in a speed vacuum concentrator for 20 min. The dried gels were rehydrated with 12.5 mg/mL trypsin in 50 mmol NH_4_HCO_3_ solution, and digestion was performed by incubation at 37 °C overnight. The digested protein samples were speed-vacuum dried and dissolved in 20 μl of water containing 0.1% formic acid, and LC‒MS/MS data were analyzed via a Hybrid FT‒ETD mass spectrometer system (Thermo Fisher Scientific).

### Histology and immunohistochemistry

The tissue samples were fixed with 4% paraformaldehyde and embedded in paraffin. Cross-sections (8 μm) were prepared and visualized via alizarin red S staining and immunohistochemistry to evaluate vascular calcification and analyze protein expression, respectively. A proximity ligation assay was performed after fixation, retrieval and permeabilization to detect binding between NEDD8 and PARP-1. The following primary antibodies were used for the immunostaining and PLA assay: NEDD8 (1:100, Cell signaling) and PARP-1 (1:100, Santa Cruz). The microscopy images were captured by an Axio Scan.Z1 scanner (Carl Zeiss Microscopy, GmbH, Jena, Germany) and a laser scanning microscope (DE/LSM700, Carl Zeiss Microscopy).

### Statistical analysis

SPSS software (version 27.0, IBM Corp, Chicago, IL) was used for the statistical analyses. Each experiment was performed in triplicate where appropriate. For two independent groups, a two-tailed unpaired Student’s t test or nonparametric Mann‒Whitney U test was applied after checking for a normal distribution. In the case of more than two groups, one-way analysis of variance (ANOVA) or two-way ANOVA with post hoc tests was used depending on the number of main effects. The data are presented as the means ± standard errors, and p values greater than 0.05 were considered statistically significant.

## Results

### MLN4924 inhibits Pi-induced calcium deposition in VSMCs

**Fig. 1:**
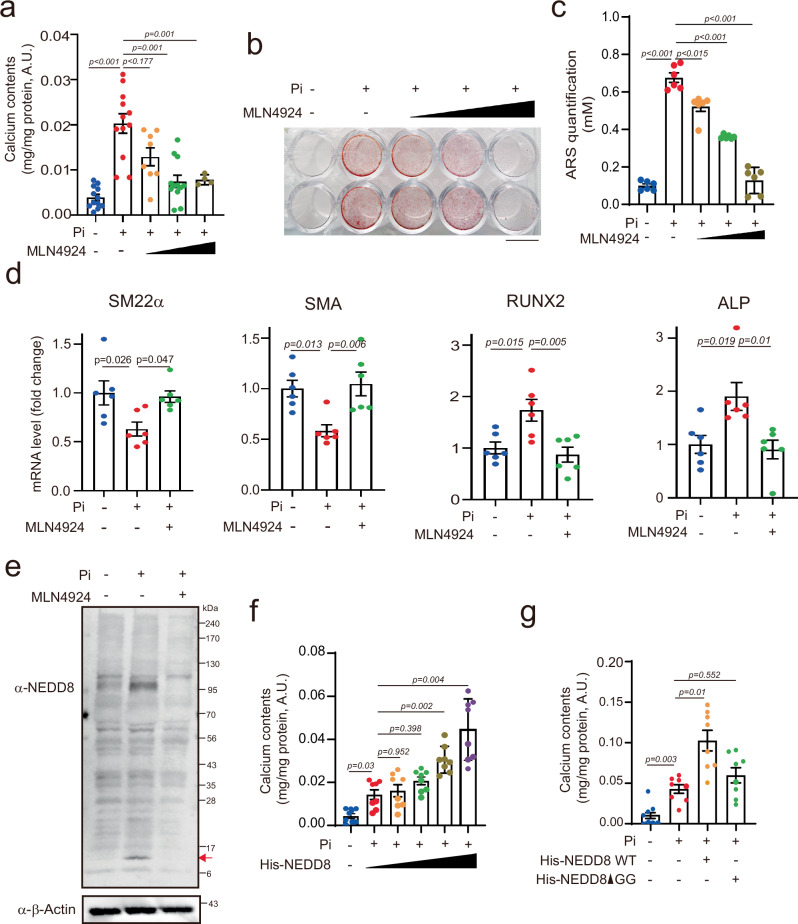
MLN4924 inhibits Pi-induced vascular calcification. **a** Treatment with MLN4924 (0.001 µM, 0.01 µM, 0.1 µM, or 1 µM) inhibited Pi (4 mM)-induced calcium deposition in A10 cells. n = 4–12 per group, independent experiments. **b** A10 cells were treated with Pi and MLN4924 under the aforementioned conditions. Representative alizarin red S-stained VSMCs showing that mineralization was blocked by MLN4924 in Pi-treated A10 cells. Scale bar, 10 mm. **c** Quantification of alizarin red S staining. n = 6 per group, independent experiments. **d** mRNA levels of smooth muscle marker genes (SM22α and SMA) and osteogenic-related marker genes (RUNX2 and ALP) were measured in A10 cells treated with Pi and MLN4924. n = 6 per group, independent experiments. **e** NEDD8 immunoblotting was performed after A10 cells were treated with Pi and MLN4924. The red arrow indicates free NEDD8. **f**, **g** The amount of accumulated calcium was measured. Overexpression of NEDD8 potentiated Pi-induced calcium deposition in A10 cells. n = 8 per group, independent experiments (**f**). Inhibition of NEDD8 conjugation with NEDD8-ΔGG blunted Pi-induced calcium deposition. n = 8 per group, independent experiments (**g**). The data are shown as the means ± SEMs. Statistical significance was tested via ANOVA with Tukey’s HSD test and Dunnett’s T3 test.

To investigate the potential involvement of NEDD8 in vascular calcification (VC), we initially assessed the effect of MLN4924 on Pi-induced calcium deposition in VSMCs. Treatment with MLN4924 resulted in a significant dose-dependent reduction in Pi-induced calcium deposition (Fig. 1a). Alizarin red S staining revealed that mineralization, a characteristic of Pi-induced VC, was effectively inhibited by MLN4924 treatment (Fig. 1b, c). Pi treatment led to a decrease in the expression of SM22α and SMA, genes associated with smooth muscle differentiation, whereas it upregulated the expression of the osteogenic genes RUNX2 and ALP. However, these changes were counteracted by MLN4924 treatment (Fig. 1d). In addition, we assessed cell viability via the MTT assay and observed that MLN4924 did not affect the viability of Pi-treated VSMCs (Supplementary Fig. 1a).

We further assessed whether Pi can induce NEDDylation via western blot analysis. The formation of poly-NEDD8 was increased in high phosphate-treated VSMCs (Fig. 1e). The overexpression of NEDD8 potentiated Pi-induced calcium deposition in VSMCs (Fig. 1f and Supplementary Fig. 1b), whereas the transfection of NEDD8-ΔGG, a mutant incapable of conjugation, inhibited Pi-induced calcium deposition (Fig. 1g and Supplementary Fig. 1c). These findings collectively suggest that the NEDD8 pathway plays a significant role in VC processes, whereas MLN4924 effectively prevents Pi-induced VC.

### NEDD8 is conjugated to PARP-1 in VC

**Fig. 2:**
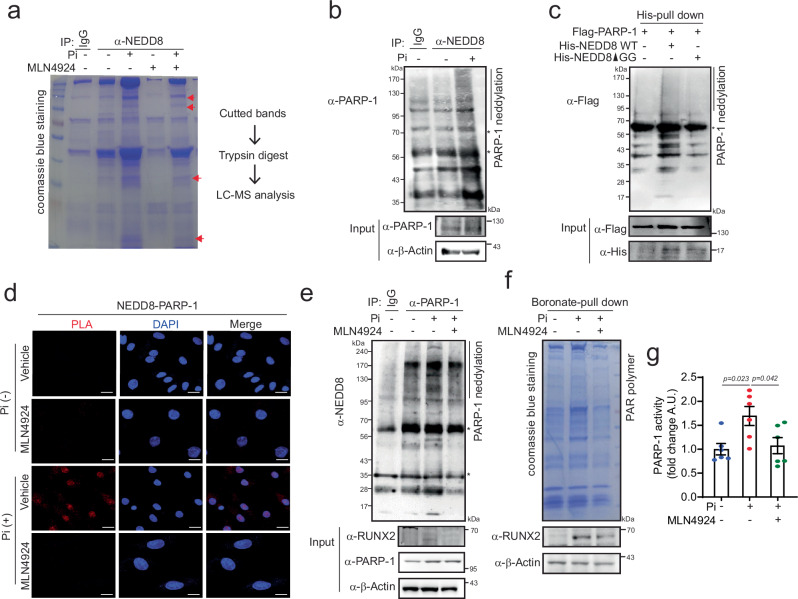
NEDD8 is conjugated to PARP-1 in VC. **a** A gel stained with Coomassie blue for mass spectrometry analysis. Cell extracts from A10 cells treated with Pi (4 mM) or MLN4924 (1 μM) were immunopurified with an anti-NEDD8 antibody and subjected to SDS‒PAGE. The bands indicated by arrows were then digested with trypsin and analyzed by mass spectrometry. **b** Cell lysates from Pi-treated A10 cells were immunoprecipitated with anti-NEDD8 and immobilized with anti-PARP-1. PARP-1 neddylation increased in Pi-induced VC. **c** Whole-cell lysates from 293 T cells transfected with the indicated constructs were subjected to Ni-NTA pull-down. PARP-1 neddylation was blunted in NEDD8-ΔGG compared with that in NEDD8 WT. **d** A PLA was performed. NEDD8-conjugated PARP-1 is dissociated by MLN4924 (1 μM). Scale bar=20 μM. **e** Immunoprecipitation with anti-PARP-1. Pi-induced PARP-1 neddylation is attenuated by MLN4924 (1 μM) in A10 cells. PARP-1 activity was measured by detecting the poly(ADP)-ribosylation (PAR) polymer (**f**) and conducting a PARP-1 enzyme activity assay (**g**). Pi-induced PARP-1 activity was blunted by MLN4924 treatment in A10 cells. n = 6 per group, independent experiments. Note: “*” indicates a nonspecific band. A.U., arbitrary units. The data are presented as the means ± SEMs. Statistical significance was tested via ANOVA with Tukey’s HSD test.

To determine which proteins undergo NEDD8-mediated modifications, we conducted immunoprecipitation-based proteomic analysis using an anti-NEDD8 antibody, followed by affinity purification and liquid chromatography‒mass spectrometry. In Pi-treated RVSMCs, NEDD8-conjugated proteins were isolated, and NEDDylation was confirmed through Coomassie blue staining. The gel bands that increased in the Pi lane and decreased in the Pi and MLN4924 lanes were removed and subjected to LC‒MS/MS to identify the NEDDylated proteins (Fig. 2a). We identified NEDD8-conjugated candidate proteins and used the PANTHER tool (PANTHERdb.org) to perform gene ontology (GO) analysis, which provided a comprehensive overview of the biological processes and molecular functions regulated by NEDDylation under VC conditions. Supplementary Fig. 2a illustrates the major cellular components, biological processes, and molecular functions. According to the enriched GO annotation, the cellular components can be divided into two categories: cellular anatomical entities and protein-containing complexes. Additionally, the identified NEDD8-conjugated candidate proteins were categorized into several major biological processes, including cellular process, biological regulation and response to stimulus, as well as diverse molecular functions, including binding, catalytic activity, and transcription regulator activity. Further bioinformatics analysis of these proteins led to the selection of candidates, including HSP90, PARP-1, EEF2, NBR1 and DDB1, as NEDD8-conjugated proteins (Supplementary Fig. 2b). We subsequently evaluated the effects of NEDD8 conjugation on calcification. The candidate proteins were effectively knocked down by siRNA (Supplementary Fig. 3a–e), and the knockdown of PARP-1 or EEF2 significantly reduced Pi-induced calcium accumulation (Supplementary Fig. 3f). Additionally, we confirmed that only PARP-1 displayed a conjugated band pattern when immunoprecipitated with anti-NEDD8 (Fig. 2b), and the other candidate proteins did not show any such pattern. (Supplementary Fig. 3g–j). Consequently, we focused on poly(ADP‒ribose) polymerase-1 (PARP-1) as a candidate protein in the context of vascular calcification. Although the roles of PARP-1 in vascular calcification are known, the significance of its posttranslational neddylation has not been extensively investigated. Therefore, we aimed to elucidate the role of neddylation of PARP-1 in the context of vascular calcification.

We next examined whether exogenous NEDD8 is conjugated to PARP-1. Overexpression of NEDD8 led to the appearance of a higher molecular weight band for PARP-1, indicative of NEDDylation. This smear pattern was abolished when NEDD8-ΔGG, a conjugation-defective mutant resulting from a Gly-75/76 deletion, was used (Fig. 2c). These findings establish that the poly-neddylation of PARP-1 with NEDD8 is intricately associated with vascular calcification.

### Visualization of the NEDD8‒PARP-1 interaction

The PLA is useful for the visualization of interacting proteins^33^. The PLA results revealed that NEDD8 binds to PARP-1 in the nucleus. However, this binding was disrupted upon treatment with MLN4924 (Fig. 2d). Furthermore, MLN4924 treatment attenuated Pi-induced PARP-1 neddylation and RUNX2 expression (Fig. 2e).

### NEDDylation of PARP-1 affects PARP-1 activity and VC

We found that PARP-1 NEDDylation was increased during vascular calcification. Given that PARP-1 is associated with vascular calcification and that its enzymatic activation involves poly (ADP)-ribosylation (PARylation)^28^, and that PARP-1 catalyzes the addition of poly (ADP‒ribose) (PAR) to substrate proteins via the cleavage of NAD^+^^34^, it is unclear whether PARP-1 NEDDylation can affect the enzymatic activation of PARP-1 in association with vascular calcification. Thus, we explored whether PARP-1 NEDDylation affects its enzymatic activity in VC. PAR polymer expression was detected by its ability to bind to boronate, as shown in Fig. 2f. Pi treatment induced PAR polymer expression, as evidenced by the smearing of boronate-conjugated precipitates (second lane). This smearing was diminished by MLN4924 treatment, which confirms its generation through NEDDylation. Cobb et al. reported that following DNA damage-induced VC, RUNX2 undergoes PARylation dependent on PARP-1 activity^35^. As shown in Fig. 2f (bottom band), RUNX2 was pulled down as a PARylated protein, which was blocked by MLN4924 treatment. Direct measurement of PARP-1 activity via a colorimetric assay (PARP universal colorimetric assay methods) revealed that Pi-induced PARP-1 activity was blunted by MLN4924 treatment (Fig. 2g). These findings underscore the dependency of PARP-1 activation on PARP-1 neddylation during the progression of VC.

### MLN4924 mitigates VD3-induced VC

**Fig. 3:**
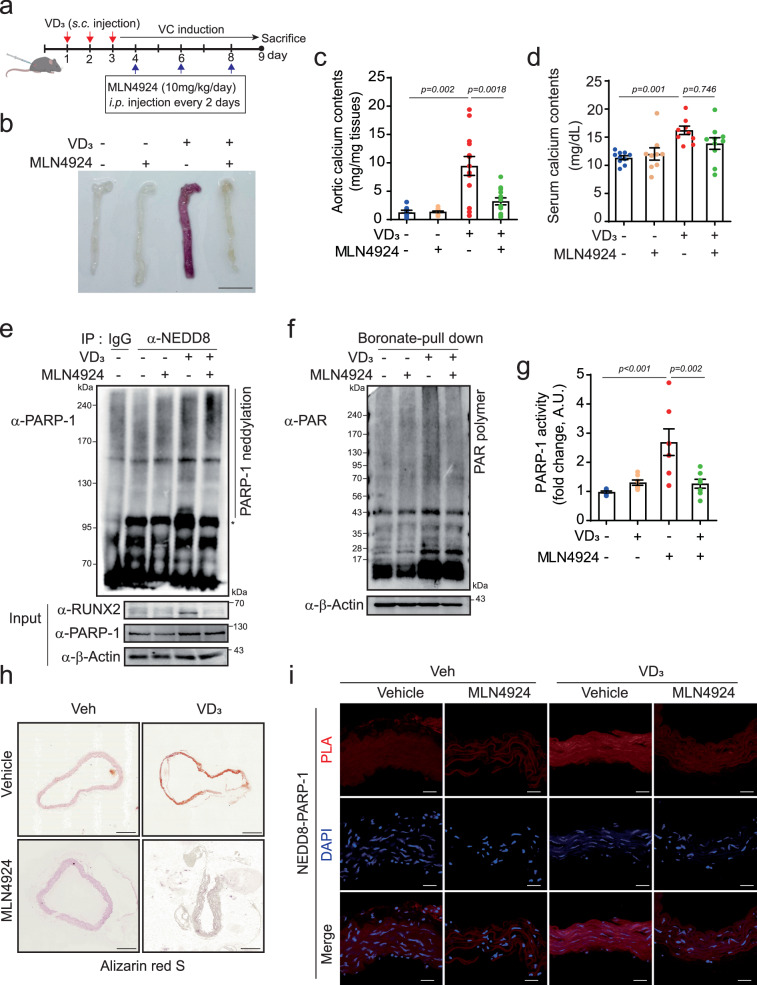
MLN4924 ameliorates VD_3_-induced VC. **a** Treatment timeline for MLN4924 (10 mg/kg/day) with VD_3_ (5 × 10^5^ IU/kg/day) in mice. **b** Whole aorta with alizarin red S staining. MLN4924 mitigated VD_3_-induced calcification. Scale bar, 10 mm. Measurement of calcium levels in the aorta (**c**) and serum (**d**) after MLN4924 treatment in VD_3_-injected mice. n = 9–11 per group, independent experiments. Treatment with MLN4924 inhibited calcium accumulation in the aorta but not in the serum of VD_3_-induced VC patients. **e** PARP-1 neddylation increased after injection of VD_3_, but was blocked by MLN4924. **f** Boronate bead-pull down assays were performed. PARP-1 activity, along with PAR polymer activity, was blunted by MLN4924. **g** Measurement of PARP-1 enzyme activity. **h** Alizarin red S staining of cross-sections of the aorta. Scale bar, 200 μΜ. **i** A PLA with anti-NEDD8 and anti-PARP-1 was performed on cross-sections. Scale bar, 20 μΜ, “*” indicates a nonspecific band. The data are presented as the means ± SEMs. Statistical significance was tested via ANOVA with the Bonferroni correction.

To evaluate the therapeutic potential of reversing NEDDylation in vascular calcification in vivo, we generated a mouse model of vitamin D_3_ (VD_3_)-induced calcification and administered intraperitoneal injections of MLN4924 (10 mg/kg) every other day for 6 days. The experimental timeline is outlined in Fig. 3a. For calcification assessment, we employed alizarin red S staining of the entire aorta. The results presented in Fig. 3b demonstrate that compared with the control treatment, MLN4924 treatment effectively counteracted VD_3_-induced VC. Calcium levels were quantified in both the arteries and serum of the mice. Remarkably, administration of MLN4924 resulted in a substantial reduction in calcium deposition within the arteries of VD_3_-treated mice, as shown in Fig. 3c. However, no significant alteration in the serum calcium level was detected in VD_3_-treated mice upon MLN4924 treatment (Fig. 3d). Importantly, both NEDD8-conjugated PARP-1 induced by VD_3_ and its enzymatic activity were effectively suppressed by MLN4924 treatment, as indicated by poly(ADP-ribose) expression (Fig. 3e, f). Additionally, MLN4924 administration attenuated PARP-1 activity in mice subjected to VD_3_ injection (Fig. 3g). As visualized by alizarin red S staining, VD_3_-induced calcium deposition appeared as reddish regions in the aortic media wall and was clearly absent after MLN4924 administration (Fig. 3h). Moreover, the interaction of PARP-1 with NEDD8 induced by VD_3_ was blunted by MLN4924 (Fig. 3i). Collectively, these findings firmly establish the efficacy of MLN4924 in ameliorating vascular calcification through the inhibition of PARP-1 NEDDylation. Furthermore, these findings underscore MLN4924 as a promising candidate for potential therapeutic interventions against VC.

### CBL-b mediates NEDD8 binding to PARP-1 in VC

**Fig. 4:**
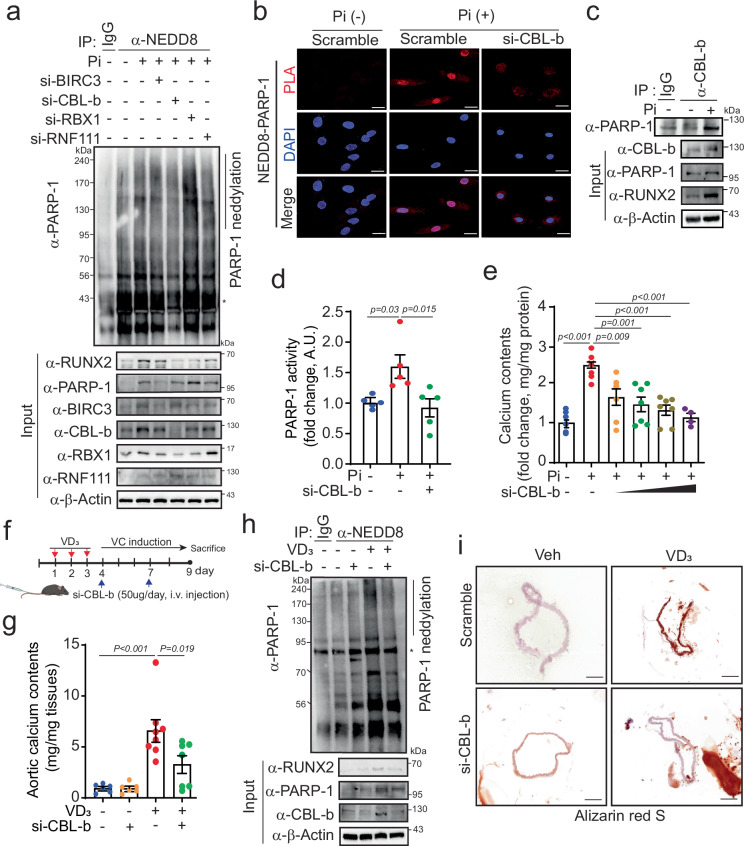
The E3 ligase CBL-b mediates PARP-1 neddylation in Pi-induced VC. **a** Immunoblot analysis of immunoprecipitates from Pi-treated A10 cells following the depletion of BIRC3, CBL-b, RBX1 and RNF111 with an anti-NEDD8 antibody. **b** A PLA was performed with anti-NEDD8 and anti-PARP-1 in A10 cells transfected with siCBL-b and Pi. Scale bar, 20 µM. **c** PARP-1 activity was analyzed in Pi-treated A10 cells with CBL-b knockdown. n = 5 per group, independent experiments. **d** Endogenous PARP-1 interacted with CBL-b in A10 cells treated with Pi. **e** Measurement of the calcium content. The knockdown of CBL-b inhibited Pi-induced calcium accumulation in a dose-dependent manner. n = 4–7 per group, independent experiments. **f** Experimental procedure timeline. The mice were treated with si-CBL-b or scramble via tail vein injection twice during the VC induction period following VD_3_ injection (5 × 10^5^ IU/kg/day). **g** Calcium assay. VD_3_-induced calcium accumulation was inhibited by CBL-b knockdown in the aorta. **h** Immunoprecipitation with an anti-NEDD8 antibody was performed. VD_3_-induced PARP-1 neddylation was blunted by CBL-b knockdown. **i** Vascular calcification in the aorta was determined by alizarin red S staining. Representative images are shown. Scale bar, 200 μΜ. “*” indicates a nonspecific band. The data are presented as the means ± SEMs. Statistical significance was tested via ANOVA with Tukey’s HSD test and the Bonferroni correction.

Given that many posttranslational modifications involve the final conjugation of small molecular moieties, facilitated by E1, E2, and E3 ligases^36^, with target specificity often guided by E3 ligases, we directed our attention toward deciphering the specific E3 ligase responsible for PARP-1 neddylation. Mammalian cells harbor several hundred E3 ligases, with CBL-3, RBX1, and FBXO11 serving as representative E3 ligases that oversee NEDDylation^12^. Consequently, we next identified the specific E3 ligase involved in PARP-1 neddylation in the context of VC. To identify dysregulated E3 ligases, we employed our previous microarray analysis (GSE74755) results for rat VSMCs treated with Pi^30^ (Supplementary Fig. 4a). Baculoviral IAP Repeat Containing 3 (BIRC3), Casitas B–lineage lymphoma protein b (CBL-b), Ring-Box 1 (RBX1), Mouse double minute 2 homolog (MDM2), Mouse double minute 4 homolog (MDM4), Ring Finger protein 7 (RNF7), and Ring Finger Protein 111 (RNF111) are dysregulated. Among these candidates, we previously reported that MDM2 mediates the ubiquitination of HDAC1 during the VC process^30^, thus we excluded MDM2 as an E3 ligase for the neddylation process of PARP-1. Next, we quantified the changes in the mRNA levels of the dysregulated E3 ligase genes in Pi-induced VSMCs via quantitative real-time PCR. Notably, BIRC3, CBL-b, RBX1, and RNF111 were significantly upregulated (Supplementary Fig. 4b). We subsequently assessed the effect of siRNA-mediated knockdown of these E3 ligases on PARP1 neddylation. Notably, only the deletion of CBL-b effectively attenuated PARP-1 neddylation, whereas the loss of other E3 ligases, such as BIRC3, RBX1, and RNF111, had negligible effects on PARP-1 neddylation (Fig. 4a and Supplementary Fig. 5a). The PLA results further reveal the dissociation of NEDD8 from PARP-1 upon CBL-b knockdown (Fig. 4b). Immunoprecipitation analysis confirmed the interaction between CBL-b and PARP-1 (Fig. 4c and Supplementary Fig. 5b). The expression of CBL-b was effectively knocked down in a dose-dependent manner in Pi-treated A10 cells via CBL-b siRNA (Supplementary Fig. 5c). The knockdown of CBL-b had no effect on the viability of A10 cells in the Pi group. (Supplementary Fig. 5d). Given that the neddylation of PARP-1 influences its activity in vascular calcification, we explored whether CBL-b plays a role in regulating PARP-1 activity. Indeed, CBL-b knockdown significantly dampened Pi-induced PARP-1 activity in VSMCs (Fig. 4d).

### Role of CBL-b in VC and its mechanistic insights

Because the role of CBL-b in VC has not been previously reported, we proceeded to evaluate its effects via the overexpression of HA-CBL-b in VSMCs. Intriguingly, CBL-b overexpression significantly potentiated Pi-induced calcium deposition in a dose-dependent manner (Supplementary Fig. 6a). Furthermore, the protein expression of CBL-b is increased in a time-dependent manner both in VSMCs treated with Pi (Supplementary Fig. 6b) and in VD_3_-induced VC model mice (Supplementary Fig. 6c). Notably, CBL-b knockdown significantly reduced the intensity of alizarin red S staining in Pi-induced VC (Supplementary Fig. 6d, e). Consistently, the increase in calcium deposition induced by Pi was blocked by si-CBL-b transfection (Fig. 4e). Next, we evaluated the effects of CBL-b knockdown by siRNA on VC in a mouse model. We intravenously injected 50 μg of si-CBL-b combined with a transfection agent twice during the VC induction period following three consecutive days of VD_3_ injection into the mice (Fig. 4f). The knockdown of CBL-b was successfully achieved in various tissues, including the aorta, brain, kidney, liver and lung (Supplementary Fig. 7a). This led to a reduction in VD_3_-induced calcium accumulation in the aorta but not in the serum (Fig. 4g and Supplementary Fig. 7b). Additionally, the loss of CBL-b in VC resulted in blunted PARP-1 neddylation and RUNX2 expression (Fig. 4h). As anticipated, the mineralization of vascular smooth muscle from the aorta in the VD_3_ group was inhibited following the injection of si-CBL-b (Fig. 4i). Collectively, these results underscore the pivotal role of the E3 ligase CBL-b in mediating PARP-1 neddylation and its regulatory role in VC.

### CBL-b E3 ligase activity governs PARP-1 neddylation in VC

**Fig. 5:**
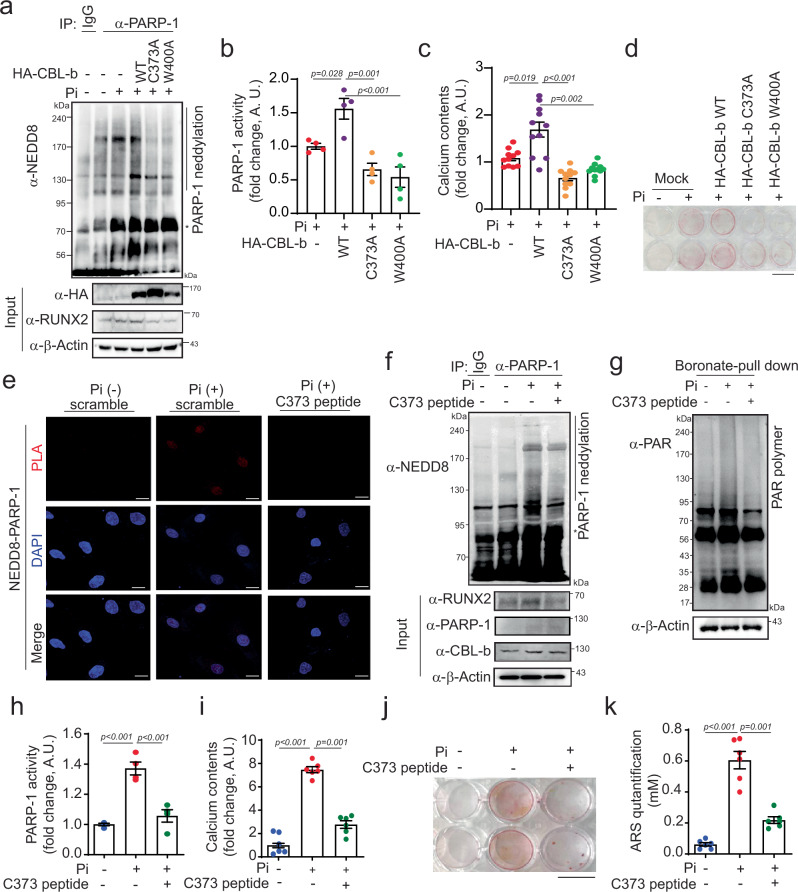
CBL-b E3 ligase activity at C373 promotes PARP-1 neddylation in Pi-induced VC. **a** PARP-1 neddylation in Pi-treated A10 cells is dependent on CBL-b E3 ligase activity. **b** PARP-1 activity was determined via an anti-PARP-1 antibody. Mutants with inactive CBL-b E3 ligase activity showed decreased PARP-1 activity in Pi-treated A10 cells. n = 4 per group, independent experiments. **c** Measurement of the calcium content. Pi increased calcium accumulation, but this effect was blocked by inactive CBL-b E3 ligase mutants (C373A and W400A). n = 11 per group, independent experiments. **d** Alizarin red S staining was performed. Scale bar, 10 mm. **e** PLA assay. NEDD8 was associated with PARP-1 under Pi conditions, but was dissociated by the CBL-b C373 peptide. Scale bar, 20 µM. **f** Immunoblot analysis was performed via immunoprecipitation with anti-PARP-1. **g** A boronate pull-down assay was performed to detect the PAR polymer. **h** PARP-1 activity was analyzed. Pi-induced PARP-1 activity was attenuated by CBL-b C373. n = 4 per group, independent experiments. **i** Calcium assay. CBL-b C373 inhibited Pi-induced calcium deposition in A10 cells. n = 6 per group, independent experiments. **j** Calcification was determined by alizarin red S staining. Scale bar, 10 mm. **k** Quantification of alizarin red S staining was performed. n = 6 per group, independent experiments. “*” indicates a nonspecific band. The data are presented as the means ± SEMs. Statistical significance was tested via ANOVA with Tukey’s HSD test and Dunnett’s T3 test.

Given that the CBL-b E3 ligases belong to the RING finger family, which engages E2 enzymes to facilitate substrate ubiquitination^37^, we examined whether the E3 ligase activity of CBL-b is essential for PARP-1 neddylation. It was previously reported that C373 and W400 of CBL-b are critical for its E3 ligase activity^38^. We generated these functionally inert forms of CBL-b that lack E3 ligase activity and next subjected them to immunoprecipitation with an anti-PARP-1 antibody. As anticipated, high Pi exposure potentiated PARP-1 neddylation (Fig. 5a) and, consequently, increased PARP-1 activity (Fig. 5b). These effects were pronounced upon transfection with CBL-b WT but not CBL-b C373A or CBL-b W400A. Interestingly, in contrast to CBL-b WT, both CBL-b C373A and CBL-b W400A failed to increase mineralization and calcium deposition (Fig. 5c, d). In addition, CBL-b WT and CBL-b E3 ligase activity dead mutants (C373A and W400A) did not alter the viability of Pi with A10 cells (Supplementary Fig. 8a).

Next, we investigated whether blocking E3 ligase activity via a peptide spanning crucial residues C373 and W400 could impede PARP-1 neddylation and, consequently, vascular calcification. Sequence analysis of different species revealed that the C373 and W400 sites of CBL-b are highly conserved (Supplementary Fig. 8b). To visualize the localization of these peptides, a fluorescein isothiocyanate-conjugated nuclear localization signal (NLS) sequence^39^ was added. The synthetic peptide C373, but not W400, effectively entered the nucleus in the VSMCs (Supplementary Fig. 8c). The CBL-b C373 peptide did not affect the viability of A10 cells cultured with Pi (Supplementary Fig. 8d). For the subsequent experiments, we utilized the C373 peptide. NEDD8 conjugated with PARP-1 is cleaved by treatment with the CBL-b C373 peptide in VSMCs under Pi conditions (Fig. 5e). Moreover, both PARP-1 neddylation and its activity were blunted by C373 in Pi-induced VC (Fig. 5f–h). Further corroborating these findings, the CBL-b C373 peptide significantly attenuated the propensity for calcium deposition and alizarin red S staining in Pi-treated VSMCs (Fig. 5i–k). Taken together, these observations underscore the pivotal role of CBL-b E3 ligase activity, particularly at residue C373, in promoting PARP-1 neddylation within the context of vascular calcification. Additionally, the C373-spanning peptide has emerged as a potential candidate for preventing vascular calcification, suggesting novel therapeutic avenues for its prevention or treatment.

### Alleviation of VD3-induced VC through the CBL-b C373 peptide

**Fig. 6:**
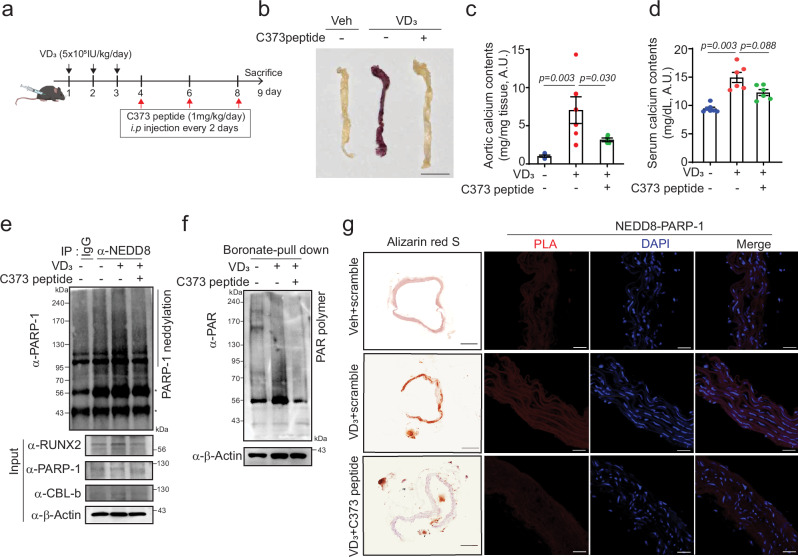
Administration of the CBL-b C373 peptide mitigated VD_3_-induced VC. **a** Experimental procedure for administering the C373 peptide (1 mg/kg/day) via intraperitoneal injection to mice with VD_3_-induced VC. **b** Alizarin red S staining of the entire aorta in the mice. Scale bar, 10 mm. Aortic calcium (**c**) and serum calcium (**d**) levels were measured. Administration of the C373 peptide reduced VD_3_-induced calcium accumulation. n = 6 per group, independent experiments. **e** Immunoprecipitation with an anti-NEDD8 antibody was used to detect PARP-1 neddylation. PARP-1 neddylation was diminished by administration of the C373 peptide to the aortas of VD_3_-induced VC mice. **f** PARP-1 activity was detected by the PAR polymer with a boronate pull-down assay. The C373 peptide blunted VD_3_-induced PARP-1 activity. **g** Histology: alizarin red S staining (left panel scale bar, 200 μΜ) and PLA with anti-NEDD8 and anti-PARP-1 (right panel, scale bar, 20 μΜ) were performed on cross-sections. VD_3_ increased vascular calcification, but was inhibited by the C373 peptide. The administration of the CBL-b C373 peptide led to the dissociation of NEDD8 from PARP-1. “*” indicates a nonspecific band. The data are presented as the means ± SEMs. Statistical significance was tested via ANOVA with Tukey’s HSD test and Dunnett’s T3 test.

To further validate the therapeutic potential of the CBL-b C373-blocking peptide, we examined its effects in VD_3_-induced VC models. The mice were intraperitoneally administered the CBL-b C373 peptide (1 mg/kg/day) every 2 days, followed by a single injection of VD_3_. After the 9-day experimental period, the mice were sacrificed, and the effects of VC were assessed (Fig. 6a). The uptake of the C373 peptide into the mouse aorta was observed through FITC fluorescence, as shown in Supplementary Fig. 9. Compared with scramble treatment, treatment with the CBL-b C373 peptide effectively attenuated the formation of calcified nodules in the aorta of VD_3_-induced VC patients (Fig. 6b). Calcium content analysis demonstrated that increased calcium deposition induced by VD_3_ was significantly counteracted by CBL-b C373 peptide treatment in the aorta but not in the serum (Fig. 6c, d). Notably, the enhancement of PARP-1 neddylation and PARP-1 enzyme activity, such as that of the PAR polymer, promoted by VD_3_ was effectively mitigated by the CBL-b C373-blocking peptide within the aorta compared with the scramble control in mice (Fig. 6e, f).

Evidently, the distinct calcium deposition characteristic of VD_3_ was entirely abrogated following the administration of the CBL-b C373 peptide (Fig. 6g, left panel). Additionally, the typical interaction between PARP-1 and NEDD8 elicited by VD_3_ was efficiently hindered by the CBL-b C373 peptide, as illustrated in Fig. 6g (right panel). Taken together, these findings underscore the efficacy of the CBL-b C373-blocking peptide in VD_3_-induced vascular calcification through the disruption of PARP-1 neddylation.

### PARP-1 neddylation is counteracted by NEDP-1 in VC

**Fig. 7:**
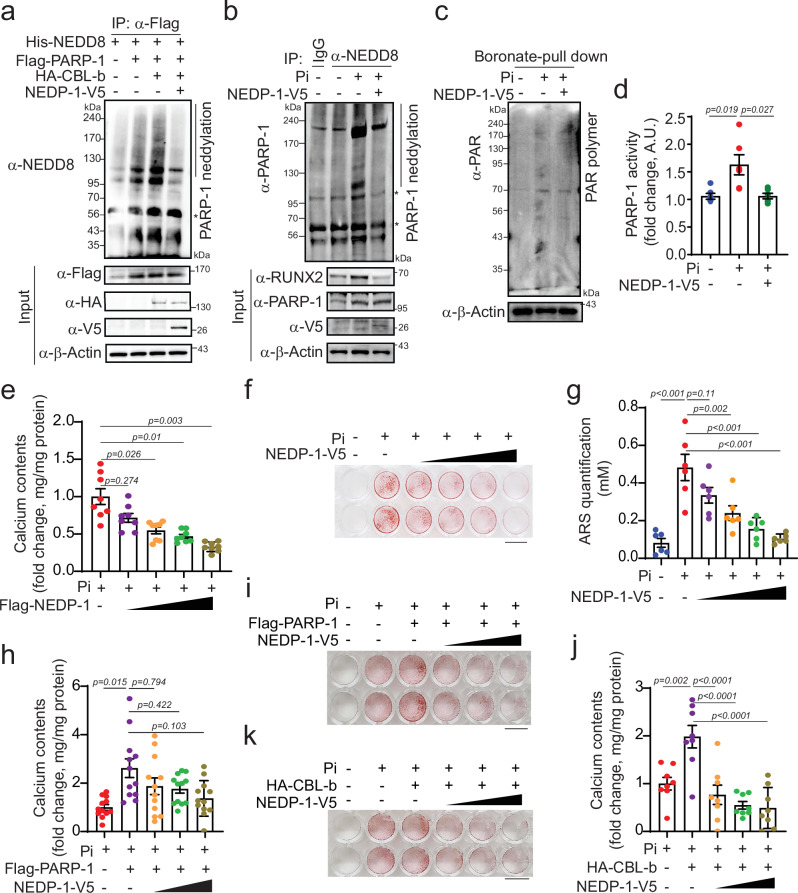
PARP-1 deneddylation by NEDP-1 attenuates VC. **a** Cell lysates extracted from 293T cells transfected with the indicated plasmid DNAs were subjected to immunoprecipitation with anti-Flag. Overexpression of NEDP-1 resulted in the denaturation of PARP-1. **b** Transfection of NEDP-1 into A10 cells followed by treatment with Pi blocked Pi-induced PARP-1 neddylation. **c** Overexpression of NEDP-1 and Pi treatment in A10 cells led to decreased expression of the PAR polymer. **d** PARP-1 activity was measured under the same conditions. n = 5 per group. **e** Measurement of the calcium content. n = 8 per group, independent experiments. **f** Cells were stained with alizarin red S. A representative whole-well image is shown. Scale bar, 10 mm. **g** The extent of alizarin red S staining was quantified. n = 6 per group. **h** Pi-treated A10 cells were cotransfected with Flag-PARP-1 and NEDP-1-V5. Measurement of the calcium content. PARP-1 potentiated Pi-induced calcium deposition, which was blocked by the overexpression of NEDP-1. n = 12 per group, independent experiments. **i** Alizarin red S staining was performed under the same conditions. Scale bar, 10 mm. **j** A10 cells were cotransfected with HA-CBL-b and NEDP-1-V5. CBL-b also potentiated Pi-induced calcium deposition, which was blocked by NEDP-1 overexpression. n = 8 per group, independent experiments. **k** Alizarin red S staining under the same conditions. Scale bar, 10 mm. “*” indicates a nonspecific band. The data are presented as the means ± SEMs. Statistical significance was tested via ANOVA with Tukey’s HSD test and Dunnett’s T3 test.

The dynamic nature of NEDDylation is well established and involves a reversible process facilitated by NEDP-1, an NEDD8-specific protease 1^40^. Therefore, we investigated whether NEDP-1-mediated de-NEDDylation could effectively reverse PARP-1 neddylation in VC. Thus, we designed a study centered on the NEDP-1. As anticipated, the introduction of ectopically expressed CBL-b augmented the conjugation of NEDD8 with PARP-1. Nevertheless, this enhancement was nullified by the overexpression of NEDP-1 (Fig. 7a). To gain deeper insights, we investigated the effects of NEDP-1 on PARP-1 neddylation and poly (ADP)-ribosylation in Pi-induced VSMCs. Notably, the overexpression of NEDP-1 resulted in the suppression of Pi-induced PARP-1 neddylation and poly(ADP)-ribosylation (Fig. 7b, c). Furthermore, the increase in the activity of PARP-1 evident in the Pi-induced VC model was effectively downregulated by the overexpression of NEDP-1 (Fig. 7d).

Until now, the role of NEDP-1 in vascular calcification has not been reported. Therefore, we sought to investigate this phenomenon. The expression of NEDP-1 gradually decreased during vascular calcification both in vitro and in vivo (Supplementary Fig. 10a, b). NEDP-1 overexpression did not affect the viability of VSMCs (Supplementary Fig. 10c). Notably, transient overexpression of NEDP-1 in VSMCs via transfection significantly affected Pi-induced calcium deposition in a dose-dependent manner (Fig. 7e and Supplementary Fig. 10d) and mineralization in VSMCs (Fig. 7f, g). Conversely, depletion of NEDP-1 significantly increased the accumulation of calcium stimulated by Pi in a dose-dependent manner (Supplementary Fig. 10e, f).

Given the function of NEDP-1 in dissociating NEDD8 from target proteins, we postulated that NEDP-1 might exert an antagonistic effect against the functional interplay of neddylated PARP-1 and the E3 ligase activity of CBL-b in the context of VC. Given this supposition, we investigated whether the functionality of PARP-1 or CBL-b could be effectively counteracted by NEDP-1. Evidently, the overexpression of either PARP-1 or CBL-b led to an increase in the calcium content and mineralization in the presence of Pi in VSMCs. Notably, this exacerbation was significantly dampened by the simultaneous overexpression of NEDP-1 in a dose-dependent manner (Fig. 7h-k). Collectively, these findings underscore the role of NEDP-1 as a pivotal counteractive element against PARP-1 neddylation in VC.

## Discussion

**Fig. 8:**
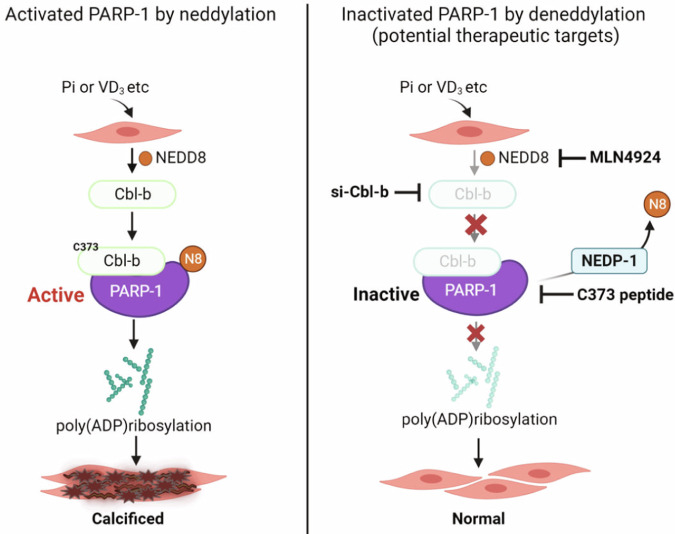
Illustration of how PARP-1 activity, facilitated by NEDD8-conjugated PARP-1, induces vascular calcification. A diverse range of stimuli, such as inorganic phosphate (Pi) or VD_3_, drive NEDD8 conjugation with PARP-1, which is mediated by CBL-b, and this process is reversed by NEDP-1. Notably, neddylation of PARP-1 leads to an increase in PARP-1 activity, which contributes to the progression of vascular calcification (VC). However, the NEDD8-activating E1 enzyme inhibitor MLN4924 effectively impedes the progression of VC. Additionally, a C373 peptide derived from CBL-b shows promise in preventing VC by mitigating the E3 ligase activity of the inactive form of CBL-b. Therefore, we propose that targeting the NEDD8-dependent activation of PARP-1 could be a potentially effective therapeutic approach for VC. Created with BioRender.com.

Here, we describe how the NEDD8 modifier is increased in response to vascular calcification stimuli and is conjugated with PARP-1, promoting vascular calcification. PARP-1 neddylation is mediated by the E3 ligase CBL-b and is deneddylated by NEDP-1. Consequently, inhibiting PARP-1 neddylation could be a potential therapeutic strategy for alleviating vascular calcification (Fig. 8).

Posttranslational modifications (PTMs) play crucial roles in various biological processes, including vascular calcification. RUNX2, a major transcription factor, can undergo diverse PTMs, such as phosphorylation, acetylation, ubiquitination and PARylation, under genotoxic stress conditions during vascular calcification. We also reported that E3 ligase MDM2-mediated HDAC1 ubiquitination induces vascular calcification^30^. Both MSX1 and MSX2 act as upstream transcription regulators of MDM2^31^. Numerous studies have reported that PARP-1 undergoes diverse posttranslational modifications, such as sumoylation, ubiquitination and acetylation^41–43^. In this study, we define neddylation as a novel modification of PARP-1 and a critical regulatory mechanism for vascular calcification. To identify neddylation substrates, some criteria are needed for the characterization of NEDD8 substrates^6^. Herein, we provide a series of data to prove that PARP-1 is a substrate for NEDD8: (i) NEDD8 is covalently attached to PARP-1; (ii) the detection of PARP-1 neddylation is endogenous; (iii) PARP-1 neddylation depends on an activating enzyme (E1), and MLN4924 inhibits PARP-1 neddylation; we identified (iv) CBL-b as a specific ligase for PARP-1 neddylation; (v) NEDP-1 as a deneddylase for PARP-1 in vivo; (vi) the formation of a polyneddylation chain on PARP-1 by NEDD8; and that (vii) PARP-1 neddylation regulates PARylation downstream. On the basis of these findings, we conclude that during vascular calcification, PARP-1 is subjected to neddylation.

To date, several researchers have studied the correlation between PARP-1 and vascular calcification. Wang et al. reported that PARP-1 could promote the osteogenic transition of VSMCs via the JAK2/STAT3/miR-204/RUNX2 pathway^29^. Other studies have demonstrated that oxidative DNA damage is a key driver of vascular calcification and that PARP-1 is activated at the site of such calcification^44^. Although, PARP-1 is activated by various stimuli and conditions associated with vascular calcification, the regulation of PARP-1 in this context is poorly understood. Here, we provide evidence that PARP-1 is conjugated with NEDD8, which in turn activates PARP-1 activity. This activation subsequently induces PARylation and promotes vascular calcification. Nonetheless, it remains unclear which lysine residues on PARP-1 bind to NEDD8. These findings necessitate further investigation into this aspect of PARP-1 neddylation activation.

CBL-b is a member of the CBL family of proteins, which consists of three homologs known as c-CBL, CBL-b, and CBL-3. CBL-b is expressed predominantly in T-cell and macrophage plaques. Studies have reported that CBL-b regulates both innate and adaptive immune cell responses through immune T-cell activation^45,46^. In atherosclerosis, genetic deficiency of CBL-b aggravated atherosclerosis in ApoE-/- mice by recruiting CD8^+^ T cells to plaques^47,48^. In this study, we found that CBL-b is upregulated and acts as a specific key mediator of PARP-1 neddylation in vascular calcification. This process depends on the E3 ligase catalytic activity of CBL-b. Catalytic inhibitors, such as those that overexpress the plasmid construct of the inactive mutant CBL-b C373A and the blocking peptide targeting residue 373 (C373), inhibit the PARP-1 neddylation mediated by CBL-b. Furthermore, inhibition of the E3 ligase catalytic activity of CBL-b hampers calcium accumulation and mineralization during vascular calcification progression. Therefore, we propose that CBL-b C373-blocking peptides may be therapeutic agents for vascular calcification. In addition, other upregulated E3 ligases, such as BIRC3, RBX1, and RNF111, do not affect PARP-1 neddylation but may serve as regulators of vascular calcification.

In conclusion, this study suggests that CBL-b acts as a critical mediator of NEDD8-conjugated PARP-1 neddylation during vascular calcification. PARP-1 neddylation plays a modulatory role in PARP-1 neddylation-mediated PARylation by regulating the catalytic activity of PARP-1. Therefore, treatment with inhibitors of PARP-1 neddylation, such as MLN4924 and CBL-b C373 blocking peptides, can block PARylation, thereby ameliorating vascular calcification. Our findings provide insights into the prevention and treatment of a variety of cardiovascular diseases related to vascular calcification.

## Supplementary information

Supplementary Information
