# Supplementary material for: CBL-b E3 ligase-mediated neddylation and activation of PARP-1 induce vascular calcification
Source: Exp Mol Med. 2024 Oct 1;56(10):2246–59. doi: 10.1038/s12276-024-01322-y (PMC11541702; doi:10.1038/s12276-024-01322-y)
Supplement: Supplementary file 1 — Supplementary Information [file 12276_2024_1322_MOESM1_ESM.docx]

**Supplementary Information (SI)**

**CBL-b E3 ligase-mediated neddylation and activation of PARP-1 induce vascular calcification**

Duk-Hwa Kwon*^1,2,3^, Sera Shin^1,2^, Yoon Seok Nam^4^, Nakwon Choe^1,2^, Yongwoon Lim^1,2^, Anna Jeong^1,2,3^, Yun-Gyeong Lee^1,2^, Young-Kook Kim^3,5^, and Hyun Kook*^1,2,3^

^1^Department of Pharmacology, Chonnam National University Medical School, Hwasun, Jeollanamdo, 58128, Republic of Korea

^2^Basic Research Laboratory for Vascular Remodeling, Chonnam National University Medical School, Hwasun, Jeollanamdo, 58128, Republic of Korea

^3^BK21 plus Center for Creative Biomedical Scientists, Chonnam National University, South Korea

^4^Department of Otolaryngology-Head and Neck Surgery, Chonnam National University Medical School and Chonnam National University Hospital, Gwangju 61469, Republic of Korea

^5^Department of Biochemistry, Chonnam National University Medical School, Hwasun, Jeollanamdo, 58128, Republic of Korea

**Supplementary Figures**

**
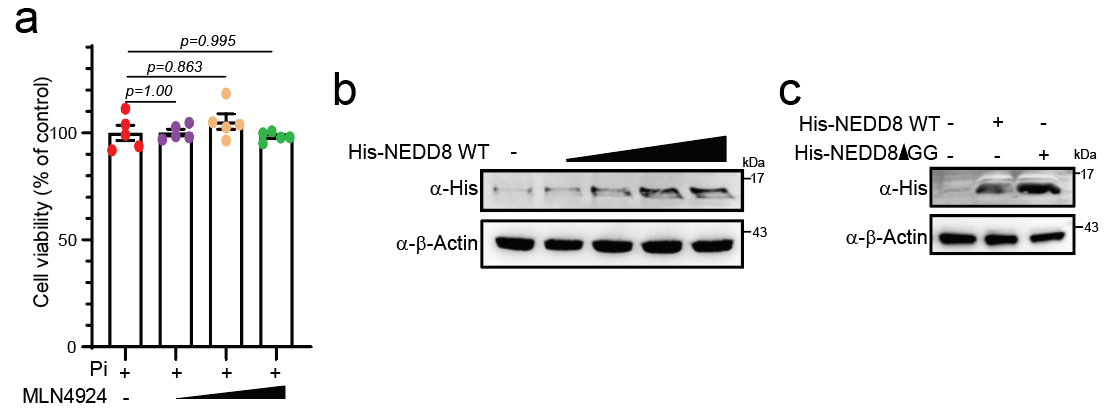
**

**Supplementary Fig 1. MLN4924, an E1 inhibitor of NEDD8, prevents Pi-induced vascular calcification.** (a) Pi-treated cells were treated with MLN4924 (0.1, 1, 10uM) for 3 days, followed by an MTT assay. The results showed that MLN4924 did not alter cell viability in Pi-treated A10 cells. N=5 per group. (b) The cells were transfected with His-NEDD8 WT (0.5, 1, 2, 4 μg) and immunoblotting was performed to assess the expression level of His-tagged NEDD8. (c) The cells were transfected with His-NEDD8 WT and His-NEDD8ΔGG, and the expression levels were examined using immunoblotting with anti-His. Data are shown as mean ± SEM. Statistical significance was tested using two-way ANOVA.

**
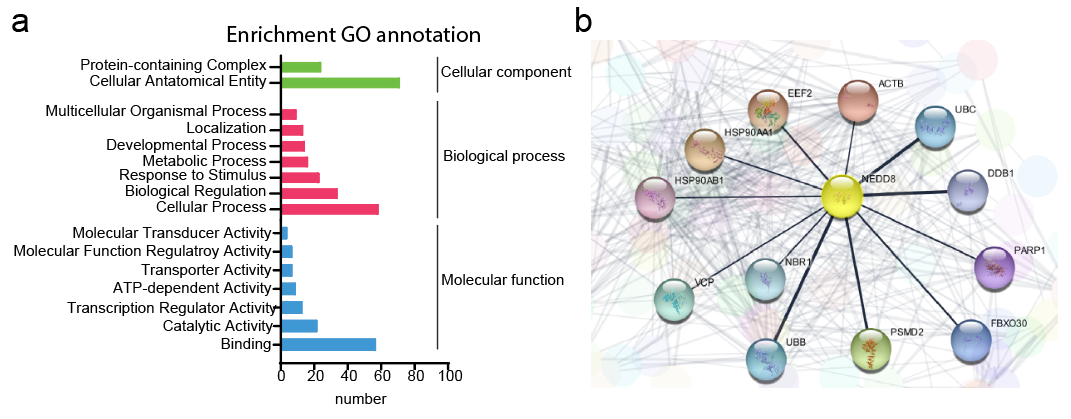
Supplementary Fig 2. Gene Ontology (GO) analysis of NEDD8-conjugated proteins in Pi-induced VC.** (a) The enriched GO categories of potential candidate proteins identified by LC-MS/MS, including cellular components, molecular functions, and biological processes, were analyzed using PANTHER DB tool. (b) STRING network displays the NEDD8-conjugated candidate proteins by STRING 12.0 and generated by Cytoscape 3.10. tool in Pi-treated VC. Abbreviation: ACTB, Actin, cytoplasmic 1; UBC, Polyubiquitin-C; DDB, DNA damage-binding protein 1; FBXO30, F-box only protein 30; PARP-1, Poly [ADP-ribose] polymerase 1; PSMD2, 26S proteasome non-ATPase regulatory subunit 2; UBB, Polyubiquitin-B; NBR1, Next to BRCA1 gene 1 protein; VCP, Transitional endoplasmic reticulum ATPase; HSP90AA1, Heat shock protein HSP 90-alpha; HSP90AB1, Heat shock protein HSP 90-beta; EEF2, Elongation factor 2.

**
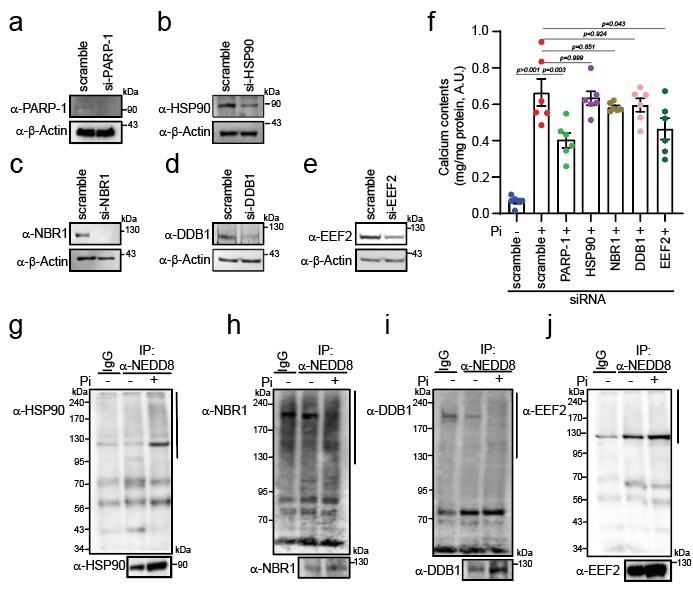
**

**Supplementary Fig 3. NEDD8-conjugated candidate proteins were analyzed in Pi-induced VC.** (a-e) A10 cells were treated with scramble, PARP-1, HSP90, NBR1, DDB1 or EEF2 siRNA for 48 hours prior to harvesting. Cell lysates were analyzed by immnublotting for the indicated candidate proteins. (f) Calcium content was determined. Among candidate proteins, knockdown of PARP-1 or EEF2 blunted the Pi-induced calcium deposition in A10 cells. n=6. (g-j) Pi-treated cell lysates were immunoprecipitated with anti-NEDD8, and the resulting SDS-PAGE-loaded membrane was probed with indicate antibodies. Abbreviation: HSP90, Heat shock protein HSP 90; NBR1, Next to BRCA1 gene 1 protein; DDB, DNA damage-binding protein 1; EEF2, Elongation factor 2. Data are shown as mean ± SEM. Statistical significance was tested using two-way ANOVA.

**
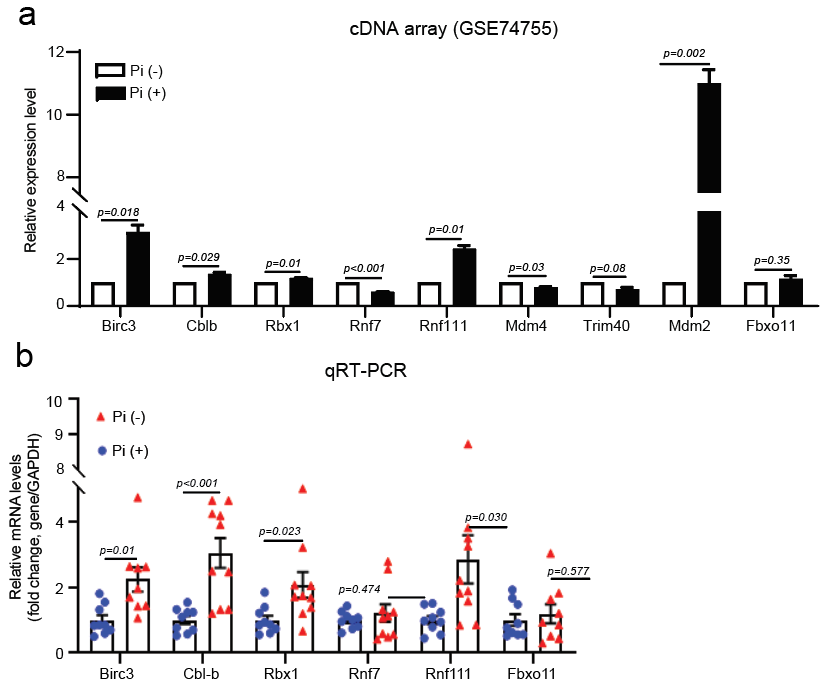
Supplementary Fig 4. The expression of E3 ligase candidate genes for neddylation was dysregulated in Pi-induced VC.** (a) Microarray analysis revealed that BIRC3, CBL-b, RBX1, RNF7, and MDM2 were upregulated in the Pi-with group (black) compared to the Pi-without group (white). n=2 per group. (b) The expression of microarray genes was validated by qRT-PCR, with GAPDH used as a normalizer. n=8~10 per group. Blue circle; without Pi; red triangle: with Pi. Data are shown as mean ± SEM. Statistical analysis is indicated. Abbreviations: BIRC3, Baculoviral IAP Repeat Containing 3; CBL-b, Casitas B–lineage lymphoma protein b; RBX1, Ring-Box 1; MDM2, Mouse double minute 2 homolog; MDM4, Mouse double minute 4 homolog; RNF7, Ring Finger protein 7; Trim40, tripartite motif-containing protein 40; RNF111, Ring Finger Protein 111; FBXO11, F-box protein 11. Statistical significance was teste using two-tailed unpaired Student’s t-test.

**
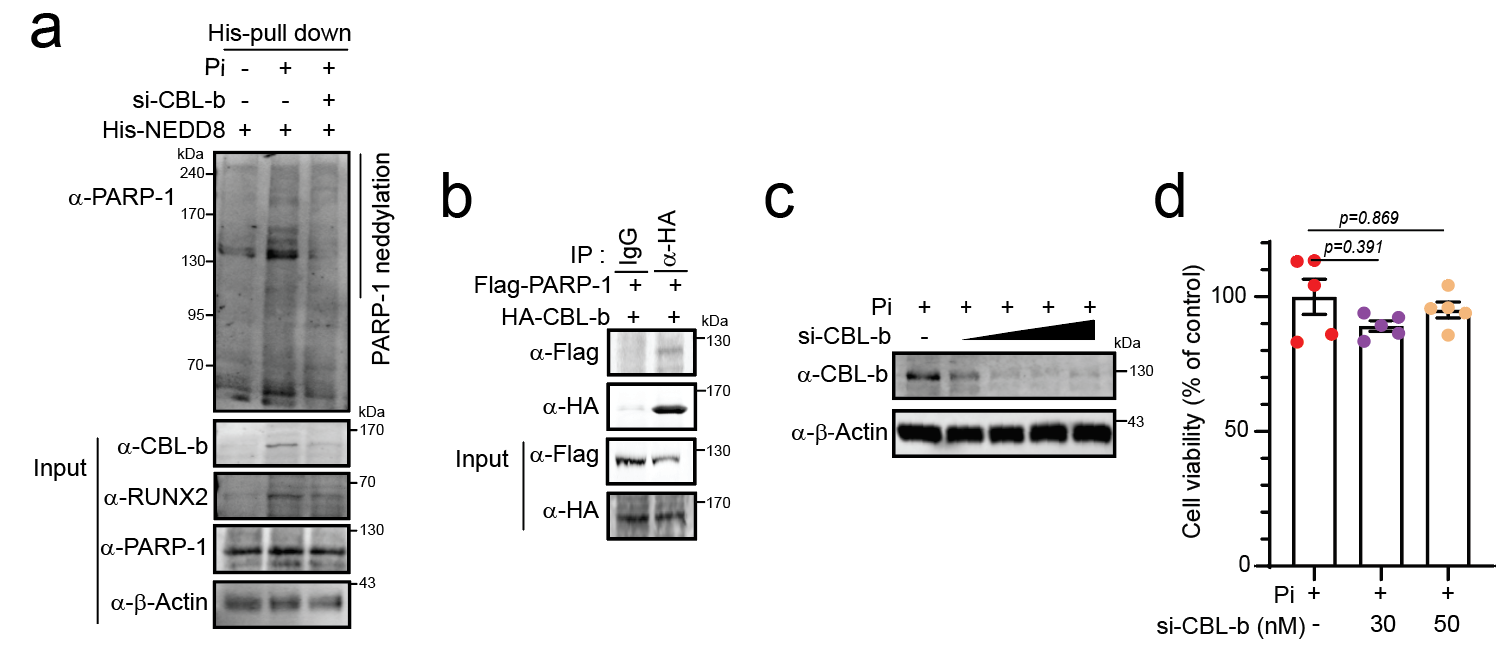
Supplementary Fig 5. Loss of CBL-b alleviates Pi-induced VC.** (a) The A10 cells were transfected with si-CBL-b and pulled down with anti-His to detect PARP-1 neddylation. Pi-induced PARP-1 neddylation is blunted by knockdown of CBL-b. (b) Exogenous interaction PARP-1 and CBL-b were performed. (c) Transfection of CBL-b siRNA in Pi-treated A10 cells resulted in a dose-dependent decrease in CBL-b expression (d) knockdown of CBL-b did not change cell viability in Pi-treated A10 cells. MTT assay was performed. n=5 per group. Data are shown as mean ± SEM. Statistical significance was tested using two-way ANOVA


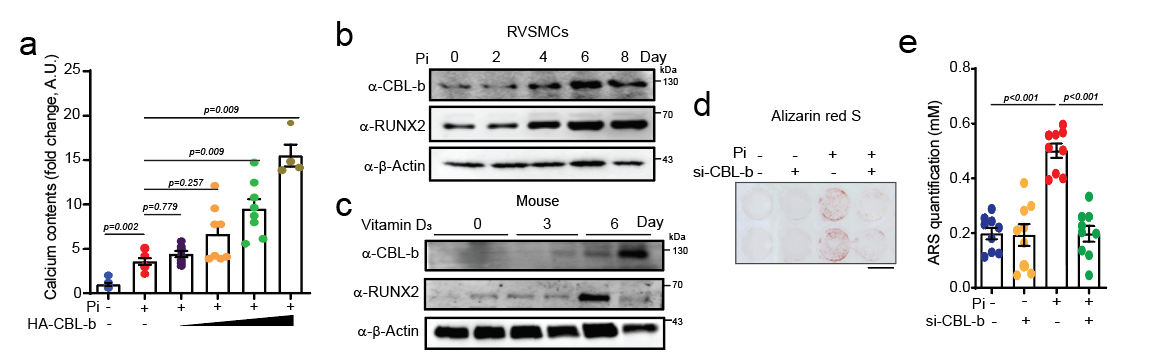


**Supplementary Fig 6. CBL-b acts as a pro-calcifying factor in Pi-induced VC.** (a) Calcium contents were measured in a dose dependent overexpression of CBL-b transfection and Pi treatment with A10 cells. Overexpression of CBL-b potentiated dose-dependently Pi-increased calcium accumulation. n=4~7 per group. (b-c) CBL-b protein expression is up-regulated during process of VC both in vitro (b) and in vivo (c). (d) Alizarin red S stained well. Depletion of CBL-b curbed on Pi-induced mineralization. Scale bar: 10mm. (e) Alizarin red S stained cells were quantified. n=8 per group. Data are shown as mean ± SEM. Statistical significance was tested using two-way ANOVA.

**
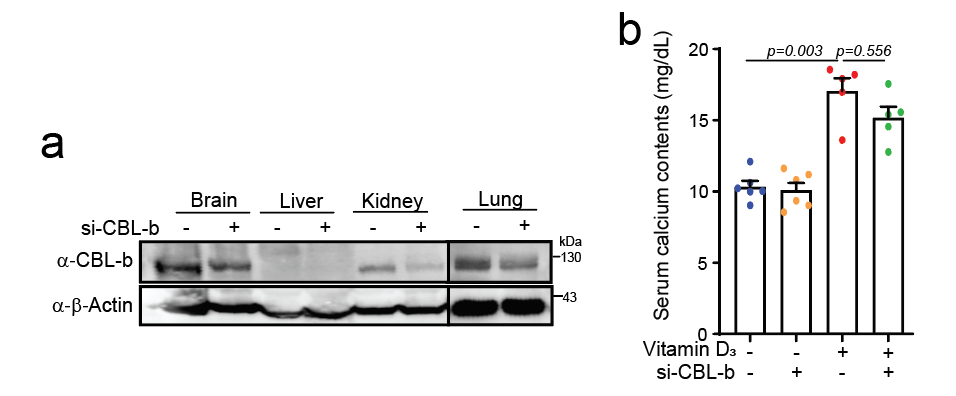
**

**Supplementary Fig 7. Knockdown of CBL-b prevents vitamin D_3_-induced VC.** (a) si-CBL-b or scramble were tail vein injection and then expression of CBL-b was checked in diverse tissues such as brain, liver, kidney, and lung. (b) Serum calcium levels were measured. n=5 per group. Data are shown as mean ± SEM. Statistical significance was tested using two-way ANOVA.

**
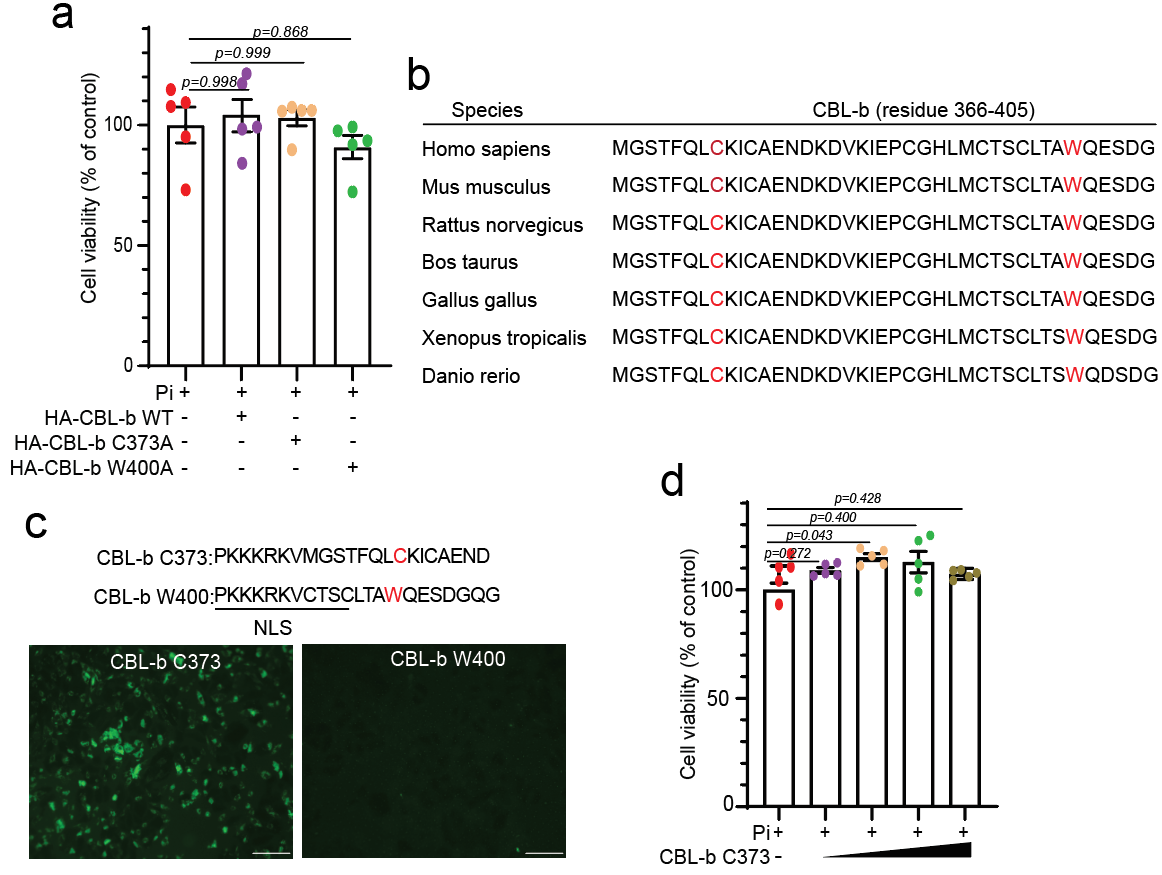
Supplementary Fig 8. Cysteine 373 at CBL-b is required for PARP-1 neddylation in VC.** (a) MTT assay revealed that cell survival was not changed either CBL-b WT or CBL-b activity dead mutants (C373A and W400A) in Pi-treated A10 cells. n=5 per group. Data are shown as mean ± SEM. (b) The conservation of the C373 and W400 site of CBL-b was analyzed. (c) Generation of fluorescein isothiocynate (FITC)-conjugated peptide CBL-b C373 and CBL-b W400 including nuclear localization signals (NLS). The cells were treated with either C373 or W400 and visualized using microscope (x200). Scale bar, 100μM. (d) The cell viability was measured. CBL-b C373 did not alter cell viability in Pi-treated A10 cells. n=5 per group. Data are shown as mean ± SEM. Statistical significance was tested using two-way ANOVA.

**
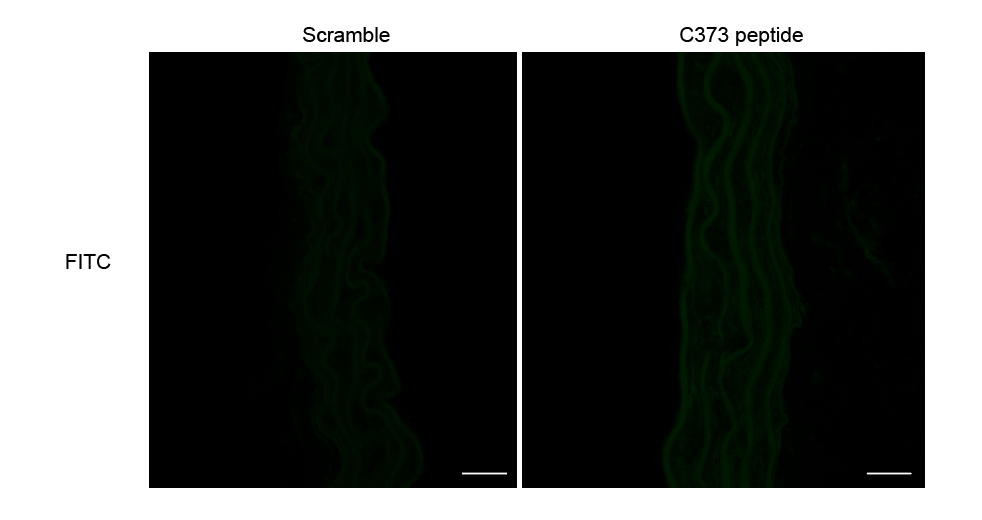
 Supplementary Fig 9. Confocal microscopy images shown the uptake of fiuorescein isothiocynate (FITC)-conjugated peptides.** Vitamin D_3_-injected mice were intraperitoneal administrated with FITC-labed C373 (1mg/kg/day) and scrambled peptide for 2 times during VC induction period. Uptake of FITC-conjugated C373 peptide was visualized using laser scanning confocal microscope (x400). Scale bar, 20uM.

**
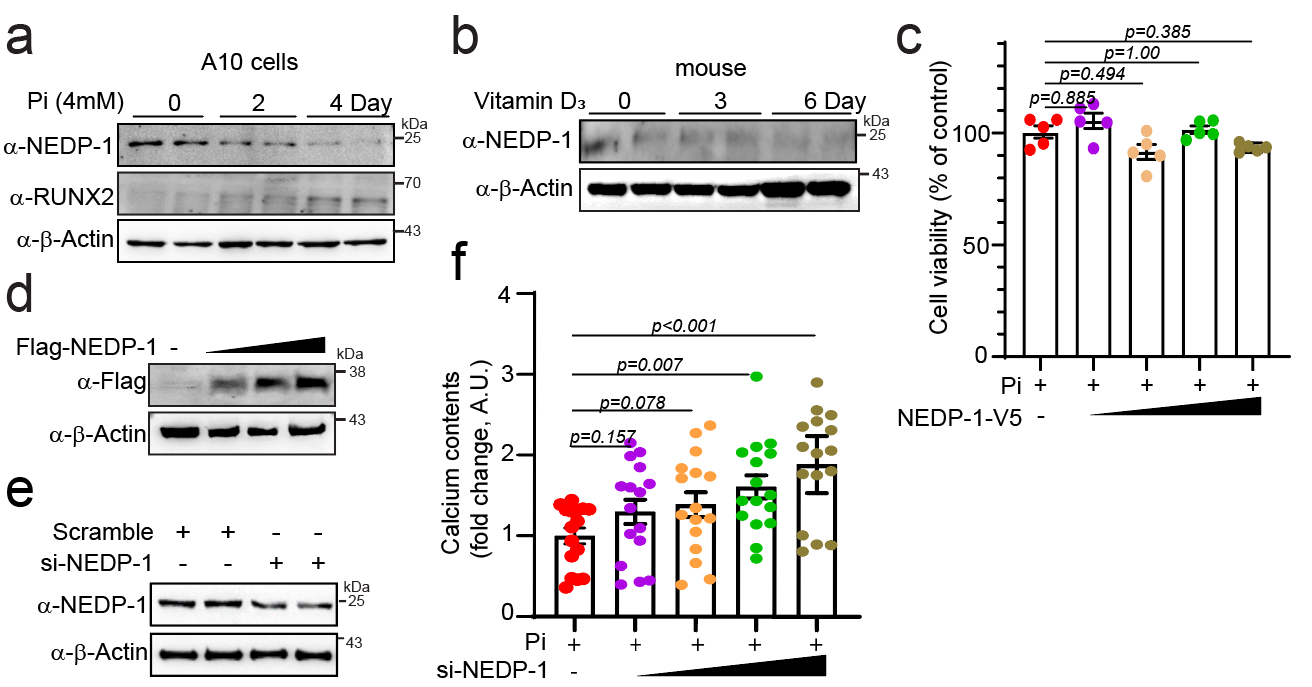
Supplementary Fig 10. NEDP-1 Decouples NEDD8 Conjugation with PARP-1 in VC. (**a) The expression of NEDP-1 decreases in a time-dependent manner in Pi-treated A10 cells. (b) In the Vitamin D3-induced VC mouse model, the expression of NEDP1- reduces over time. (c) Cells treated with Pi were transfected with NEDP-1-V5 (50, 100, 200, 400ng) and cell viability was measured using the MTT assay. NEDP-1 did not alter cell viability in Pi-induced VC. n=5 per group. (d) Overexpression of NEDP-1 post-transfection with Flag-NEDP-1 was confirmed by Flag expression using immunoblotting in A10 cells. (e) Knockdown of NEDP-1 was achieved by transfecting A10 cells with si-NEDP-1. (f) Calcium assay in A10 cells with si-NEDP-1 (10, 30, 50, 100nM) transfection and Pi treatment. Loss of NEDP-1 dose-dependently potentiated Pi-induced calcium deposition in A10 cells. n=12 per group. Data are shown as mean ± SEM. Stasticcal signicance was teste using ANOVA.

**Supplementary Materials and Methods**

**Reagents**

Antibodies against anti-RUNX2 (1:1000, ab23981) and EEF2 (1:1000, ab75748) were from Abcam (Abcam, Cambridge, UK); NEDD8 (1:1000, #2745) and HSP90 (1:1000, #79641) were from Cell signaling (Cell signaling technology, Inc., Danvers, MA, USA); Flag (1:1000, F7425 and F1804), NEDD8 (1:1000, SAB4501973) and HA (1:1000, H9658) were from Sigma (Sigma-Aldrich Corp., St. Louis, MO, USA); PARP-1 (1:500, sc8007), CBL-b (1:200, sc8006), Poly(ADP)ribose (1:500, sc56198), RBX1 (1:1000, sc-393640), MDM2 (1:1000, sc8017), NBR1 (1:500, sc130380), DDB1 (1:500, sc376860), His (1:1000, sc8036), and beta actin (1:1000, sc-47778) were from Santa Cruz Biotechnology (Santa Cruz Biotechnology, Inc., Santa Cruz, CA, USA); NEDP-1 (1:1000, NBP1-31208) was from Novus (Novus Biologicals, Centennial, CO, USA**)**. Anti-mouse (1:5000, 7076S) or anti-rabbit (1:5000, 7074S) IgG peroxidase-conjugated secondary antibodies were purchased from Sigma (Sigma-Aldrich).

Cholecalciferol (vitamin D_3,_ C9756), PDD00017273 (PARG inhibitor, SML1781) and m-Aminophenylboronic acid-agarose (boronate bead, A8312) were purchased from Sigma (Sigma-Aldrich Corp., St. Louis, MO, USA). MLN4924 was purchased from Calbiochem (EMD Millipore). *CBL-b* siRNA, *NEDP-1* siRNA, *BIRC3* siRNA, *RBX1* siRNA, *RNF111* siRNA, *HSP90* siRNA, *NBR1* siRNA, *DDB1* siRNA, *EEF2* siRNA and scramble were purchased from Bioneer (Daejeon, Korea).

**Synthesis of the CBL-b blocking peptide**

The decoy peptide containing CBL-b C373 or CBL-b W400 was derived from the CBL-b protein sequence surrounding the Cys 373 or Trp 400 site. To facilitate uptake into cells, the decoy peptide was conjugated to a nuclear localization signal (NLS, PKKKRKV). The peptides (purity, >90%; peptron, Daejeon, Korea) were resuspended in double-distilled water at a stock concentration of 6 mM and incorporated into A10 cells at a final concentration of 10 μM for 3 days. The peptide was replenished every 2 days when culture media was changed.

**Plasmids**

*Flag-NEDP-1* was purchased from Addgene (Watertown, MA, USA). *Flag-PARP-1 WT, His-NEDD8 WT,* and *His-NEDD8 ΔGG* were kindly provided by Prof. Ho Jin You (Chosun University, Gwangju, Korea). *HA-CBL-b* was kindly provided by Prof. Nacksung Kim (Chonnam National University, Gwangju, Korea. *pCEFL-HA-CBL-b C3730A*, and *pCEFL-HA-CBL-b W400A* were constructed by site-directed mutagenesis (CosmoGeneTech, Seoul, Korea) based on *pCEFL-HA-CBL-b WT. pcDNA3-NEDP-1-v5* was constructed by sub-cloning based on *pcDNA3.1-v5.* All plasmids were checked by direct sequencing before use.

**Cell cultures**

Rat vascular smooth muscle cells (RVSMCs) were isolated from thoracic aorta of 6~8-week-old Sprague-Dawley male rats after CO_2_ inhalation, following a previously described method(Kwon *et al*, 2016) . RVSMCs cultured in 10% fetal bovine serum (FBS; Hyclon) in DMEM (GIBCO) with antibiotics and were used at passages 2 to 7. A10 cells derived from embryonic rat aorta were purchased from American Type Culture Collection (ATCC, CRL-1476, Manassas, VA, USA) and were maintained in 10% FBS in DMEM with antibiotics. Human embryonic kidney 293T cells were obtained from the Seoul Korean Cell Line Bank (KCLB, 21573, Seoul, Korea) and were maintained in 10% FBS in DMEM with antibiotics. All cells were incubated in a humidified atmosphere with 5% CO_2_.

**Total RNA preparation and Quantitative Real-time PCR**

Total RNA was extracted using TRIzol Reagent (Invitrogen, Waltham, MA, USA) according to the manufacturer’s instructions. The cDNA synthesis was generated using iScript™ cDNA Synthesis Kit (Bio-Rad Laboratories, Inc., Hercules, CA, USA) and analyzed by quantitative real-time polymerase chain reaction (qRT-PCR) using a QuantiTech SYBR Green RT- PCR Master Mix (QIAGEN, Valencia, CA, USA) and a Rotor gene Q (Qiagen, Hilden, Germany). All data were normalized to GAPDH. Relative mRNA levels were quantified using the 2^-ΔΔCt^ method. To rule out possible genomic DNA contamination, primers were designed to include an intervening intron. Information of primers for qRT-PCR was as follows: rat CBL-b, forward, 5’-GGA CAG ACG GAA TCT CAC AAA-3’, and reverse, 5’-GGA AGT TAT CTC CCT GGA ACT G-3; rat Birc3, forward, 5’-CTG TAC CGC TTG TCC ACA TAT T-3’, and reverse, 5’-GGT AGG GAC TTG TGC TGA AAG-3’; rat Fbxo11, forward, 5’-TGC ATC CTG AAC CTG GTA AAT-3’, and reverse, 5,-CGT CCT CAG AAC CTT CCA TAA A , rat Mdm4, 5’- AAC ATC TGG TGT ACT GTG GTG-3’, and reverse 5’- TGC TCT GGA ATC TCT GCA TTT A-3’; rat Rbx1, forward, 5’-GGC GAT GGA TGT GGA TA-3’, and reverse, 5’-GAG AGA TGC AGT GGA AAT G-3’, rat Rnf7, forward, 5’-TCC TTT CCT CGC ATT CT-3’, and reverse, 5’-CAC AAC ACA GTC CTC TTG-3’; rat Rnf111, forward, 5’-GAG ACA TTC CCA GCC TAT-3’, and reverse, 5’-CTC TCC TTC TGC TTC TGT-3’; rat Trim140, forward, 5’-GGA CAA CCA GGA CAT TTG-3’, and reverse, 5’-TGC TAG ACT CAC AGA ACC-3’; rat, Nedp-1, forward, 5’-CCT GAA GTC ACC CAG TTC ATT A-3’, and reverse, 5’-CGG CTG CCT GGT TAG AAT TA-3’; rat SM α actin, forward, 5’- AGT CGC CAT CAG GAA CCT CGA GAA-3, and reverse, 5,- GCC AGA TCT TTT CCA TGT CGT CCC-3’; rat SM22α r, forward, 5,- CCC ACA AAC GAC CAA GCC TTT TCT-3’, and reverse, 5’- CCT GTT CCA TCT GCT TGA AGA CCA-3’; rat GAPDH, forward, 5’-TGC ACC ACC AAC TGC TTA G-3’, and reverse, 5’-GAT GCA GGG ATG ATG TTC-3’, and purchased from Bioneer (Korea). Pre‐designed qPCR primers for rat RUNX2 (Rn01512298_m1) and ALP (Rn00677879_m1) were purchased from Thermo Fisher (Waltham, MA USA).

**Immunoprecipitation and Western blot analysis**

Cells and tissues were lysed with 0.5% NP-40 solution including 50 mM Tris (pH 8.0), 150 mM NaCl, 1 mM EDTA, 1 mM DTT, 1 mM phenylmethylsulfonyl fluoride, 1 mM Na_3_VO_4_, and 1 μg/mL each of leupeptin, pepstatin, and aprotinin. One milligram of proteins was then immunoprecipitated overnight at 4 °C with the indicated antibody, following adding A/G bead and incubation for 1 hour. After extensive washing with lysis buffer, the immunocomplexes were analyzed by western blotting assay. The lysates were separated by SDS-PAGE and transferred overnight at 120 mA onto a PVDF (Millipore, Bedford, MA, USA) and blocked with 5% skim milk in 1x TBST. Membranes were incubated with the specific primary antibodies overnight at 4°C. After 3 washes in 1x TBST, membranes were incubated with horseradish peroxidase-linked secondary antibodies for 1 hour at RT. Membranes were again washed 3 times in 1x TBST, and protein bands were visualized by enhanced chemiluminescence using Western Blotting Luminol Reagent (Santa Cruz) and a Fusion FX7 imaging system (Vilber, Marne-la-vallée, France).

**MTT assay**

The cells were plated in 96-well plates and treated with MLN4924. After 48 hours, the cells were treated with 10 μL 3-(4,5-dimethylthiazol-2-yl)-2,5-diphenyltetrazolium bromide (MTT, Sigma-Aldrich) and incubated for 2 hours at 37℃ and then changed to 100 μL dimethyl sulfoxide (DMSO). Absorbance was measured at 570 nm by using an ELx808 absorbance reader (BioTek Instruments).

**Appendix References**

Kwon, D.H. *et al.* MDM2 E3 ligase-mediated ubiquitination and degradation of HDAC1 in vascular calcification. *Nat. Commun.* **7**, 10492 (2016)
